# Dementia-related death statistics in Korea between 2013 and 2023

**DOI:** 10.12771/emj.2025.00304

**Published:** 2025-03-31

**Authors:** Seokmin Lee

**Affiliations:** Statistics Research Institute, Statistics Korea, Daejeon, Korea

**Keywords:** Cause of death, Alzheimer disease, Vascular dementia, International Classification of Diseases, Republic of Korea

## Abstract

**Purpose:**

This study aimed to analyze dementia-related death statistics in Korea between 2013 and 2023.

**Methods:**

The analysis utilized microdata from Statistics Korea’s cause-of-death statistics. Among all recorded deaths, those related to dementia were extracted and analyzed using the underlying cause-of-death codes from the International Classification of Diseases, 10th revision.

**Results:**

The number of dementia-related deaths increased from 8,688 in 2013 to 14,402 in 2023. The crude death rate rose from 17.2 per 100,000 in 2013 to 28.2 per 100,000 in 2023, although the age-standardized death rate declined from 9.7 to 8.7 over the same period. The dementia death rate is 2.1 times higher in women than in men, and mortality among individuals aged 85 and older exceeds 976 per 100,000. By specific cause, Alzheimer’s disease accounted for 77.1% of all dementia deaths, and by place, the majority occurred in hospitals (76.2%), followed by residential institutions including nursing homes (15.3%) in 2023.

**Conclusion:**

The rising mortality associated with dementia, especially Alzheimer’s disease, highlights a growing public health concern in Korea. These findings support the need for enhanced prevention efforts, improved quality of care, and targeted policies addressing the complexities of dementia management. It is anticipated that this empirical analysis will contribute to reducing the social burden.

## Introduction

### Background

Dementia is recognized as a major public health problem worldwide, and its prevalence is increasing as the population ages. In Korea, Alzheimer’s disease—a primary form of dementia—rose from the 10th leading cause of death in 2013 to the 6th in 2023, representing 3.2% of all deaths [1]. Additionally, the annual cost of Alzheimer’s disease treatment in Korea is approximately 2.3 trillion won [2]. The World Health Organization defines dementia as an umbrella term for several diseases that affect memory, cognition, and daily functioning [3]. Because dementia primarily affects the elderly, it leads to memory loss, cognitive decline, and behavioral disturbances. This not only imposes a heavy disease burden on patients but also significantly affects their families, making dementia both a public health and a social issue.

The study of dementia-related deaths is essential for effective clinical management and informed policymaking. Understanding the characteristics and trends of these deaths provides critical data to improve the quality of life for patients and their families.

### Objectives

This study aimed to analyze the causes of death and related factors associated with dementia and to examine the death characteristics of dementia patients in Korea from 2013 to 2023.

## Methods

### Ethics statement

This study analyzed de-identified microdata produced by Statistics Korea; therefore, institutional review board approval or informed consent was not required.

### Study design

This descriptive study was conducted in accordance with the STROBE (Strengthening the Reporting of Observational Studies in Epidemiology) statement, which is available at https://www.strobe-statement.org/.

### Setting, participants, data source, and measurement

Microdata from Statistics Korea’s cause-of-death statistics were used to analyze dementia-related mortality rates and death characteristics. These national official statistics are compiled by analyzing death certificates and 22 types of administrative data—including health insurance records and cancer registration data—for all deceased individuals. For the dementia analysis, the International Classification of Diseases, 10th revision codes F01 (vascular dementia), F03 (unspecified dementia), G30 (Alzheimer disease), and G31 (other degenerative diseases of the nervous system, not elsewhere classified) were utilized.

### Bias

There was no bias in data collection and analysis.

### Study size

The entire population of Korea was included. No sample size estimation was required.

### Statistical methods

Descriptive statistics were employed to present the findings. Mortality was analyzed using the number of deaths, the crude mortality rate, and the age-standardized death rate—standardized to the 2005 mid-year population.

## Results

### Number of deaths and mortality rate due to dementia

**Fig. 1.**
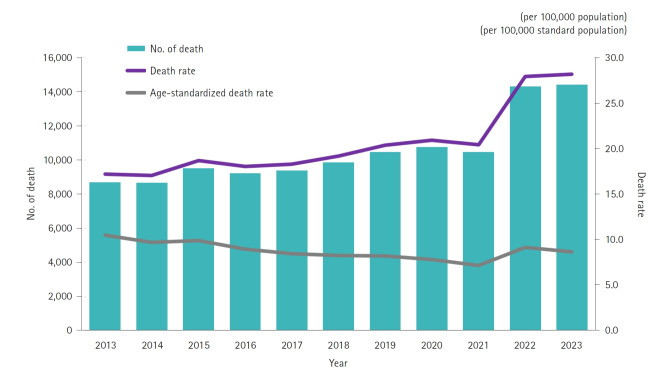
Number of deaths, death rate, and age-standardized death rate due to dementia-related diseases between 2013 to 2023 in Korea.

In 2023, Korea recorded 14,402 dementia-related deaths, with a crude mortality rate of 28.2 per 100,000 population; dementia accounted for 4.09% of all deaths. In 2013, there were 8,688 dementia-related deaths with a mortality rate of 17.2 per 100,000. Both the number of deaths and the crude mortality rate have shown an increasing trend, with the 2022 mortality rate rising by 36.7% compared to the previous year (Supplement 1). The age-standardized death rate for dementia in 2023 was 8.7 per 100,000, following a decline from 2018 to 2021 and then an increase in 2022 (Fig. 1, Supplement 2).

### Deaths due to dementia by sex and age

In 2023, dementia-related deaths in women numbered 9,737 vs. 4,665 in men. The mortality rate for women was 37.9 per 100,000—2.1 times higher than that for men (Table 1). Although the death rate remained minimal before the age of 50, it increased markedly beginning in the 70–74 age group, reaching 976.0 per 100,000 for those aged 85 and older. Mortality rates tended to rise with age. When analyzed by sex and age, the rate for men was higher until age 79, but for individuals aged 85 and older, women experienced a relatively higher rate (Table 2).

### Mortality rate by specific cause of death due to dementia

**Fig. 2.**
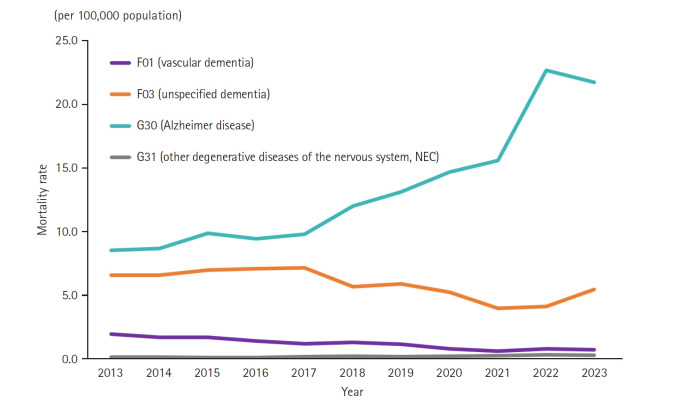
Specific causes of death among dementia-related deaths between 2013 to 2023 in Korea. NEC, not elsewhere classified.

In 2023, the mortality rate for Alzheimer’s disease was 21.7, for unspecified dementia 5.4, for vascular dementia 0.7, and for other degenerative diseases of the nervous system 0.3. Since 2013, the mortality rate for vascular dementia has continuously declined from 1.9 to 0.7, whereas Alzheimer’s disease has shown an increasing trend. In particular, the Alzheimer’s mortality rate increased significantly from 15.6 to 22.7 in 2022 (Fig. 2). Among dementia-related deaths in 2023, Alzheimer’s disease accounted for 77.1%, a notable rise from 49.7% in 2013.

### Place of death due to dementia

In 2023, most dementia-related deaths occurred in medical facilities such as hospitals (76.2%), followed by residential institutions including nursing homes (15.3%), and homes (8.1%). In contrast, the overall distribution of deaths by place was 75.4% in medical facilities, 15.5% at home, and 5.9% in residential institutions.

**Fig. 3.**
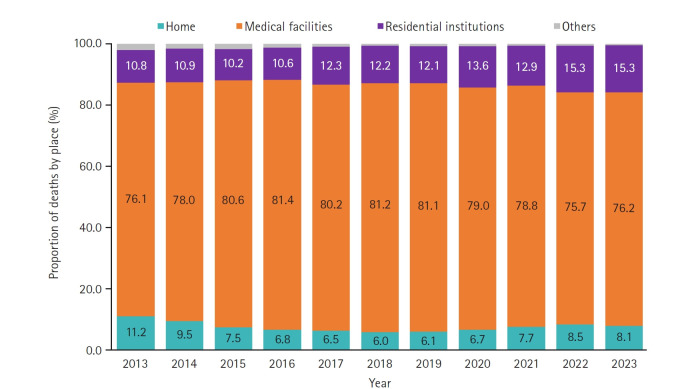
Place of death due to dementia-related diseases between 2013 to 2023 in Korea.

Since 2013, hospital deaths have declined while home deaths have increased, with a marked change in trend beginning in 2018. Deaths in residential institutions have steadily risen from 10.8% in 2013 to 15.3% in 2023. In 2022, when dementia-related deaths surged, the proportion of hospital deaths decreased from 78.8% to 75.7% (Fig. 3).

## Discussion

### Key results

In 2023, the dementia death rate was 28.2 per 100,000 population, accounting for 4.09% of all deaths. Due to an aging population, dementia-related deaths have continued to increase, with a sharp rise observed in 2022—when many deaths were attributed to coronavirus disease 2019 (COVID-19), particularly the Omicron variant of severe acute respiratory syndrome coronavirus 2. The dementia death rate is 2.1 times higher in women than in men. Alzheimer’s disease accounted for 77.1% of dementia-related deaths in 2023, with its mortality rate increasing sharply in 2022, unlike other dementia types. Additionally, most dementia-related deaths occurred in hospitals (76.2%), followed by residential institutions (15.3%), while the proportion of home deaths has been rising since 2018.

### Interpretation

The simultaneous increase in the crude death rate and the decrease in the age-standardized death rate indicate that demographic aging has significantly influenced mortality levels. The burden is disproportionately higher among women (37.9 vs. 18.3 per 100,000) and older adults, peaking at 976.0 per 100,000 for those aged 85 and older. Alzheimer’s disease is the predominant cause of dementia deaths (77.1%), with its mortality rate rising sharply, while the rate for vascular dementia is declining. Furthermore, the increased share of deaths in residential institutions (15.3%) reflects a shift in care settings. These trends underscore the need for targeted interventions—especially for aging women—and enhanced long-term care infrastructure.

#### How does dementia cause death?

Patients with dementia face an elevated risk of death not only from direct neurodegeneration but also from indirect factors such as pneumonia, malnutrition, and falls. This increased risk is linked to significant lifestyle changes as brain damage progresses. Cognitive decline and reduced physical activity contribute to difficulties with eating, personal hygiene, and medication management, while airway obstruction or aspiration pneumonia may occur from food or secretions. Ultimately, dementia acts as an underlying cause of fatal events including pneumonia, starvation and dehydration, loss of appetite, urinary tract infections, diabetes, infections related to immunosuppression, falls, and airway obstruction due to dysphagia. A previous study analyzing Korean data found that the risk of death for elderly individuals diagnosed with dementia was approximately 8.4 times higher than for those with normal cognition [4].

#### Korean health policy on dementia

The Korean government has been strengthening its support for dementia management. In 2008, it introduced the “1st Comprehensive Dementia Management Plan,” and in 2011, it enacted the Dementia Management Act. Since then, various measures related to dementia have been periodically announced. Given Korea’s robust national health insurance system, its dementia management policies emphasize long-term care services and financial support for medical expenses. Notably, dementia-related policies expanded significantly in 2018. Key initiatives included broadening the scope of nursing insurance services to cover patients with mild dementia regardless of physical function, and implementing health insurance coverage for dementia diagnostic tests to reduce patient costs. This policy expansion enabled nursing care for patients with mild dementia who did not require hospitalization, and these changes appear to have influenced the shift in the place of death since 2018.

#### Impact of COVID-19 on dementia mortality

The COVID-19 pandemic has had multifaceted impacts worldwide, with particularly severe consequences for vulnerable populations, such as dementia patients. During the pandemic, dementia-related deaths can be attributed both to direct COVID-19 infection and to indirect effects from social changes. Previous studies have shown that dementia patients are at higher risk of COVID-19 diagnosis, hospitalization, and death due to weakened immune systems [5]. In Japan, analyses revealed that both the number of dementia patients and the mortality rate increased during the pandemic, attributed to factors such as social isolation from quarantine measures and reduced availability of medical and nursing services [6]. A multivariable analysis in Korea demonstrated that the dementia group had a higher mortality risk than the non-dementia group (odds ratio, 3.05; P<0.001) among a nationwide cohort of 2,800 subjects over 50 diagnosed with COVID-19 between January and April 2020 [7]. According to the 2022 cause-of-death statistics in Korea, the crude death rate increased by 17.4% from the previous year—the largest rise since 1983—while the COVID-19 death rate increased by 522.8% [8]. These findings indicate that although the COVID-19 epidemic peaked in Korea in 2020, its impact on overall mortality was substantial in 2022.

A linear regression model fitted to data from 2013 to 2021 estimated an annual increase in the dementia death rate of approximately 0.48 per 100,000. The predicted value for 2022 (year 9, with 2013 as year 0) was around 21.3 per 100,000—lower than the observed 27.9—even before accounting for the sharp rise in 2023. Alternatively, assuming a linear increase from 2021 to 2023 (an increase of 7.8 over 2 years, or 3.9 per year), the estimate would be 20.4+3.9=24.3 per 100,000, consistent with interpolation. Given that the observed dementia death rate in 2022 was 27.9, it can be inferred that the surge in dementia-related deaths that year is linked to the COVID-19 pandemic.

### Conclusion

This study examined the demographic characteristics and major trends in dementia-related mortality. The findings provide a basis for developing policy approaches to improve the health and quality of life for dementia patients. Dementia is both a personal health issue and a significant social challenge. Consequently, research on dementia mortality is expected to contribute to alleviating the social burden by identifying preventive strategies to reduce mortality and by guiding improvements in medical services.

## Figures and Tables

**Table 1.** The number of dementia-related deaths and death rate by sex between 2013 and 2023

Year	No. of deaths (deaths)			Death rate (per 100,000 population)		
	Both sexes	Men	Women	Both sexes	Men	Women
2013	8,688	2,740	5,948	17.2	10.8	23.5
2014	8,663	2,625	6,038	17.1	10.3	23.8
2015	9,519	2,857	6,662	18.7	11.2	26.1
2016	9,223	2,883	6,340	18.0	11.3	24.8
2017	9,375	2,745	6,630	18.3	10.7	25.8
2018	9,847	3,049	6,798	19.2	11.9	26.5
2019	10,453	3,173	7,280	20.4	12.4	28.3
2020	10,747	3,396	7,351	20.9	13.3	28.6
2021	10,476	3,362	7,114	20.4	13.1	27.6
2022	14,301	4,455	9,846	27.9	17.4	38.3
2023	14,402	4,665	9,737	28.2	18.3	37.9

**Table 2.** The death rate due to dementia by sex and age group in 2023

Age group (yr)	Death rate (per 100,000 population)			Sex ratio (M/W)
	Both sexes	Men	Women	
Total	28.2	18.3	37.9	0.5
<40	0.0	0.0	0.0	1.4
40–44	0.1	0.1	0.1	1.0
45–49	0.3	0.5	0.1	8.8
50–54	0.3	0.2	0.4	0.5
55–59	1.4	2.2	0.6	3.8
60–64	3.7	4.9	2.6	1.9
65–69	8.7	12.1	5.6	2.2
70–74	21.4	28.9	14.7	2.0
75–79	60.5	71.3	52.0	1.4
80–84	212.1	230.3	200.6	1.1
≥85	976.0	809.0	1,044.2	0.8

## Supplementary materials

Supplementary files are available from Harvard Dataverse: https://doi.org/10.7910/DVN/HJWUF0

Supplement 1. The number of deaths due to dementia in Korea, 2013~2023.

Supplement 2. Data for generating Figures 1 to 3.
